# Investigating the Role of Gut-Derived Neurotoxin TMAO in PTSD Risk Following Traumatic Brain Injury

**DOI:** 10.62641/aep.v53i4.1885

**Published:** 2025-08-05

**Authors:** Dongliang He, Qin Kang, Wei Duan, Guilan Li, Renli He, Xiaoping Liu, Xianghao Gong

**Affiliations:** ^1^Department of Nutrition, Affiliated Hengyang Hospital of Hunan Normal University & Hengyang Central Hospital, 421001 Hengyang, Hunan, China; ^2^Department of Oncology, Affiliated Hengyang Hospital of Hunan Normal University & Hengyang Central Hospital, 421001 Hengyang, Hunan, China; ^3^Department of Neurosurgery, Affiliated Hengyang Hospital of Hunan Normal University & Hengyang Central Hospital, 421001 Hengyang, Hunan, China; ^4^Department of Cardiovascular, Affiliated Hengyang Hospital of Hunan Normal University & Hengyang Central Hospital, 421001 Hengyang, Hunan, China

**Keywords:** trimethylamine N-oxide, traumatic brain injury, post-traumatic stress disorder, relevance

## Abstract

**Background:**

Post-traumatic stress disorder (PTSD), comorbid with traumatic brain injury (TBI), severely affects the mood state of patients. Trimethylamine N-oxide (TMAO), one of the key intestinal flora metabolites, strongly correlates with TBI. This study aimed to explore the role of TMAO in the development of TBI-related PTSD and assess its predictive significance.

**Methods:**

This study included 120 TBI patients treated at the Affiliated Hengyang Hospital of Hunan Normal University & Hengyang Central Hospital between February 2022 and April 2024. The clinical data were obtained from the hospital's medical record system. Patients were divided into a PTSD group (n = 56) and a non-PTSD group (n = 64) based on the post-traumatic stress disorder self-rating scale (PTSD-SS). Furthermore, patients in the PTSD group were divided into mild and severe subgroups. Blood samples were collected, and serum TMAO levels were assessed. Additionally, the correlation between TMAO levels, PTSD incidence, and PTSD severity was evaluated. The risk factors for PTSD comorbid with TBI and its severity were evaluated using univariate and multivariate logistic regression analyses. Finally, receiver operating characteristic (ROC) curve analysis was performed to assess the diagnostic effectiveness of TMAO as a predictive marker for PTSD.

**Results:**

Multivariate analysis showed that female gender, lower per capita monthly household income, depression, anxiety, and higher serum TMAO levels were significant risk factors for PTSD. Depression, anxiety, and higher serum TMAO levels were associated with severe PTSD, and higher per capita monthly household income and intracranial infection were protective factors. Serum TMAO levels were significantly higher in PTSD patients than in non-PTSD patients (*p* < 0.001), with its level profoundly elevated in severe PTSD patients than in mild PTSD patients. Furthermore, the correlation analysis revealed that TMAO was positively correlated with the severity of PTSD (r = 0.8582, *p* < 0.0001). ROC curve analysis indicated TMAO's sensitivity of 67.86% and specificity of 93.75% for predicting PTSD, with an area under the curve (AUC) of 0.8175.

**Conclusion:**

Serum TMAO levels were significantly elevated in PTSD patients comorbid with TBI and were closely associated with PTSD severity. Furthermore, TMAO may aid in the early identification of high-risk, severe PTSD patients following TBI, thus helping to optimize intervention strategies.

## Introduction

Traumatic brain injury (TBI) is a common clinical condition resulting from external trauma [1], often leading to severe complications and representing a major cause of mortality and disability. Its global incidence continues to rise, affecting over 50 million individuals each year. In China, the mortality rate of TBI is approximately 13 per 100,000 individuals [2]. Primary brain injury from TBI can result in ischemic brain damage, paralysis, concussion, or even death [3] and is often complicated by various neurological disorders in the later stages [4], such as aphasia and cognitive impairment. Additionally, TBI patients usually experience a range of post-traumatic psychological stress responses [5, 6], with post-traumatic stress disorder (PTSD) being one of the most common [7].

PTSD is triggered by psychological trauma and is characterized by re-experiencing the traumatic event, avoidance behaviors, negative changes in cognition and mood, and significant alterations in arousal and reactivity [8]. It is associated with stress-related pathologies such as neuroinflammation, oxidative damage, and excitotoxicity, which lead to white and gray matter injury [9]. These mechanisms overlap with the secondary damage observed in TBI. Given its clinical significance, the association between TBI and PTSD has been extensively studied.

Investigating the role of the gut-brain axis in central nervous system diseases has been increasingly focused in the recent years. Particularly, the effect of gut microbiota and their metabolites on neuroinflammation and neurofunctional impairment after brain injury has gained attention. Numerous illnesses, such as atherosclerosis, cardiovascular disorders, and metabolic syndrome, have been linked to trimethylamine N-oxide (TMAO), a metabolite produced by the liver’s oxidation of trimethylamine (TMA), a product of gut microbes [10]. TMAO may influence the nervous system by modulating metabolic pathways in the liver and gut and by activating inflammatory responses and inducing oxidative stress. Research on the role of TMAO in brain injury and related neuropsychiatric disorders is still in its early stages. Some studies suggest that TMAO may contribute to neurodegeneration following brain injury by influencing neuroinflammatory responses [11]. However, the precise role of TMAO in the development of TBI comorbid with PTSD remains unclear.

This study aims to provide a new biomarker for the early diagnosis and intervention of PTSD following TBI by analyzing serum TMAO levels in PTSD patients. It combines multifactorial risk analysis with an assessment of TMAO’s diagnostic efficacy. Additionally, we explore the correlation between TMAO levels and the severity of PTSD to understand its role in disease progression, offering a basis for developing personalized treatment strategies.

## Research Subjects and Methods

### Research Subjects

This study included 120 TBI patients with a good prognosis who underwent surgery at the Affiliated Hengyang Hospital of Hunan Normal University & Hengyang Central Hospital between February 2022 and April 2024. Patients were enrolled within one-week post-craniotomy. The severity of the head injury was classified as open or closed based on the nature of the trauma, a distinction critical for understanding its potential impact on PTSD outcomes. The Glasgow Outcome Scale (GOS) [12] was assessed 3 months after craniotomy. Additionally, the post-traumatic stress disorder self-rating scale (PTSD-SS) [13] scores and clinical data were also collected at the same time point. The patients (n = 120) were divided into two groups, the PTSD group (n = 56) and the non-PTSD group (n = 64), based on the PTSD incidence. Furthermore, within the PTSD group, patients were divided into mild (50 ≤ PTSD-SS scores < 60) and severe (PTSD-SS scores ≥60) subgroups.

**Fig. 1.**
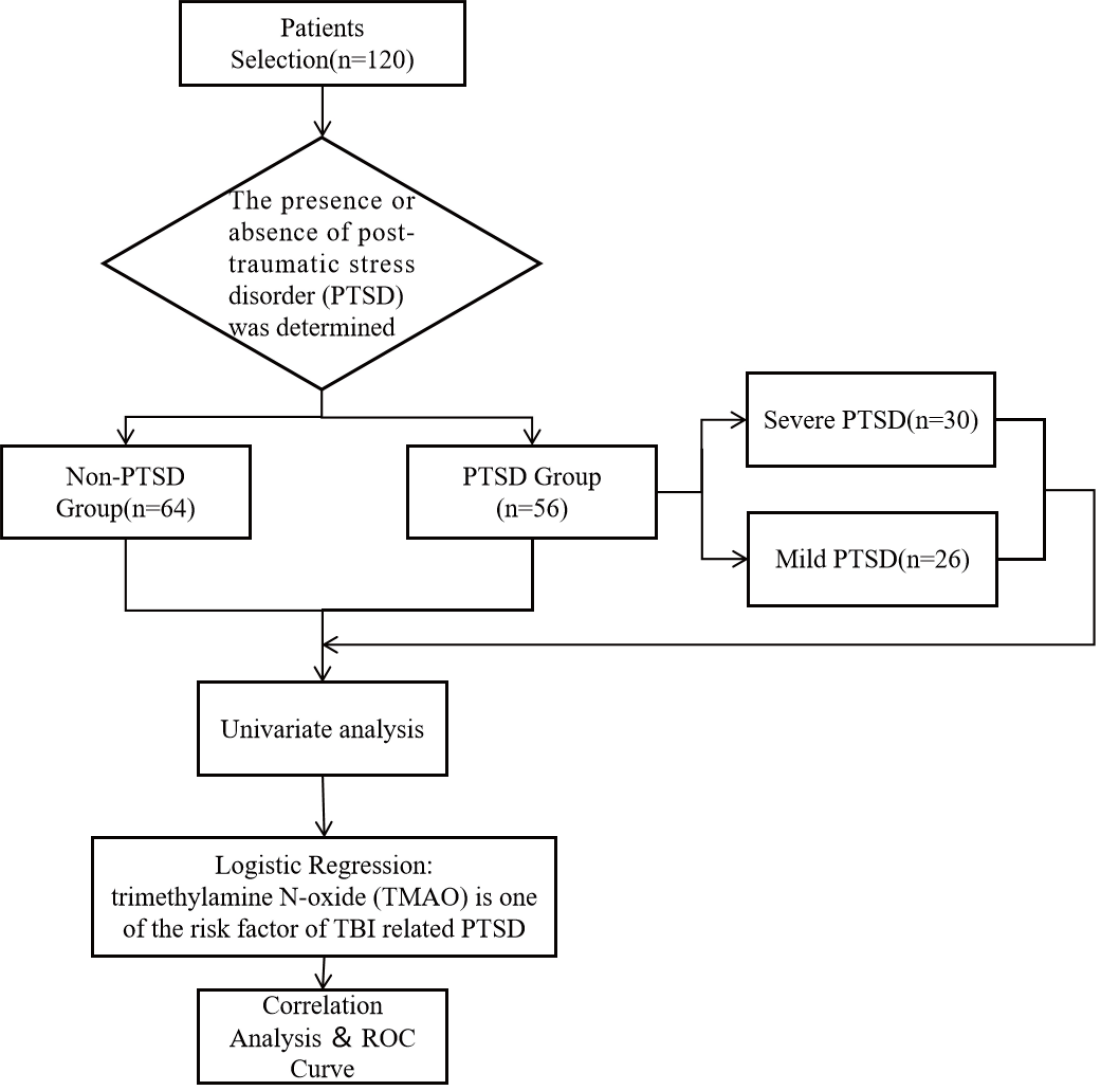
** A flowchart of patient selection and grouping**. ROC, receiver operating characteristic; TBI, traumatic brain injury.

Informed consent was obtained from all patients or their families. This study followed the principles of the Declaration of Helsinki. A flow chart of patient selection and grouping is shown in Fig. 1.

### Inclusion and Exclusion Criteria

The inclusion criteria included ① patients diagnosed with TBI using computed tomographic (CT) or magnetic resonance imaging (MRI); ② patients with GOS scores ≥4 three months after craniotomy; and ③ those aged eighteen years or above.

The exclusion criteria were as follows: ① patients with severe organic damage or dysfunction of the cardiopulmonary, liver, or kidney systems or with other endocrine diseases affect metabolism; ② patients who developed severe stress ulcers within 48 hours of admission; ③ patients with schizophrenia, mood disorders, delusional disorders, or anxiety disorders; ④ patients with incomplete clinical data or low family compliance; and ⑤ consumption of foods or medications containing probiotics, antibiotics, or steroids within the last 3 months.

### Baseline Characteristics of the Study Participants

The baseline characteristics of all patients were obtained from the hospital’s electronic medical record system. The data obtained included age, gender, body mass index (BMI), marital status, per capita monthly household income, injury factors, type of damage, financial reimbursement, primary caregiver, time from injury to first aid, and intracranial infection.

### Post-traumatic Stress Disorder Self-Rating Scale (PTSD-SS)

The PTSD-SS [13] assessment included subjective evaluations (scoring range: 1 to 5), re-experiencing experiences (scoring range: 7 to 35), avoidance symptoms (scoring range: 7 to 35), increased arousal (scoring range: 6 to 30), and impaired social functioning (scoring range: 2 to 10). A total score of ≥50 indicates the presence of positive symptoms, while a score of ≥60 suggests severe PTSD. Higher scores correspond to greater severity of PTSD.

### Self-Rating Anxiety Scale (SAS)

The SAS [14] is a widely used psychological tool for evaluating a person’s anxiety level. Comprising 20 items with an overall score of 80, it evaluates the participant’s experiences with a range of anxiety-related feelings over the past week. Each item is assessed on a 4-point Likert scale, ranging from “no anxiety” to “extremely severe anxiety”, with higher scores suggesting more severe anxiety. SAS scores for each group were recorded before and one month after the intervention. Anxiety was defined at a total score greater than 50.

### Self-Rating Depression Scale (SDS)

The SDS [15] is a widely used psychological for assessing an individual’s depression level. The scale usually comprises 20 items, with a total score of 80, and covers various facets of depression. A 4-point Likert scale is used to assess an individual’s moods over the past week, ranging from “no depression” to “extremely severe depression”, with higher scores suggesting more severe depression. SDS scores were documented for both groups before and one month following the intervention. Depression was defined as a total score greater than 53.

### Detection of TMAO

Serum TMAO levels were measured using the enzyme-linked immunosorbent assay (ELISA) method. Blood samples (5 mL) were collected from the patient’s antecubital veins the day after admission and from the control group on the morning of their health check while fasting. The samples were centrifuged at 3000 rpm for 20 minutes at 4 °C (with a centrifuge radius of 10 cm), and the supernatant was collected and stored at –20 °C until analysis. Standard solutions with varying concentrations were prepared following the instructions of the TMAO ELISA test kit (BS-9921, Bensgheng (Tian Jin) Health Technology Co., Ltd., Tianjin, China). The absorbance of the various concentrations of standard solutions at 450 nm was determined using an ELISA plate reader (HSA-W2096, Shenzhen Haisi’an Biotechnology Co., Ltd., Shenzhen, China). The serum TMAO levels were assessed using the standard regression curve analysis approach.

### Statistical Methods

Statistical analyses were performed using SPSS 20.0 (IBM Corp., Armonk, NY, USA). Continuous data were evaluated for normality using the Shapiro-Wilk test. The *t*-test was used for comparing two samples, and normally distributed continuous data were indicated as mean ± standard deviation. Group comparisons were conducted using the Mann-Whitney U test, and non-normally distributed continuous data were displayed as median (minimum, maximum). Categorical data were represented as [n (%)] and statistically analyzed using the χ^2^ test. Pearson’s correlation coefficient was used for correlation analysis. Statistical significance was set at a *p*-value of <0.05. Univariate and multivariate analyses were conducted to identify risk factors for PTSD following TBI and for PTSD severity. Furthermore, receiver operating characteristic (ROC) analysis was performed to assess the diagnostic value of TMAO in PTSD patients comorbid with TBI.

## Results

### Univariate Analysis of Risk Factors for PTSD Comorbid With TBI

Out of the total of 120 TBI patients, 64 individuals were included in the non-PTSD group and 56 in the PTSD group. Univariate analysis revealed several significant risk factors for PTSD. Gender was significantly associated with PTSD (*p* < 0.001), with a higher number of females in the PTSD group. Low monthly household income (<$275) was linked to an elevated risk of PTSD (*p* = 0.003), as was the absence of financial reimbursement (*p* = 0.011). Furthermore, intracranial infection (*p* = 0.008), depression (*p* < 0.001), and anxiety (*p* < 0.001) were strongly associated with PTSD. A higher proportion of PTSD patients had unrelated caregivers (*p* = 0.036). However, no significant differences were found in age (*p* = 0.418) or BMI (*p* = 0.177). Depression and anxiety were significantly more common in PTSD patients (*p* < 0.001 for both), indicating their critical role in PTSD development (Table 1).

**Table 1.** **Univariate analysis of risk factors for PTSD comorbid with TBI [$\bar{x}$ ± s, n (%)]**.

Variables		Non-PTSD group	PTSD group	*t*/$\chi^{2}$	*p*-value
N		64	56		
Gender	Male	44 (68.75)	20 (35.71)	13.096	<0.001
	Female	20 (31.25)	36 (64.29)		
Age (years)		45.19 ± 5.34	45.91 ± 4.26	0.812	0.418
BMI (kg/m^2^)		23.00 ± 2.62	22.32 ± 2.89	1.358	0.177
Marital status	Married	35 (54.69)	35 (62.50)	0.750	0.386
	Unmarried, divorced and	29 (45.31)	21 (37.50)		
Per capita monthly household income	<$275	24 (37.50)	36 (64.29)	8.571	0.003
	≥$275	40 (62.50)	20 (35.71)		
Injury factors	Traffic accident	24 (37.50)	25 (44.64)	0.872	0.832
	Fall down	20 (31.25)	15 (26.79)		
	Violence	14 (21.87)	10 (17.86)		
	Other	6 (9.38)	6 (10.71)		
Type of damage	Closed head injury	32 (50.00)	26 (46.43)	0.153	0.696
	Open head injury	32 (50.00)	30 (53.57)		
Financial reimbursement	Yes	40 (62.50)	22 (39.29)	6.445	0.011
	No	24 (37.50)	34 (60.71)		
Primary caregiver	Custody of kin	52 (81.25)	36 (64.29)	4.395	0.036
	Unrelated care	12 (18.75)	20 (35.71)		
Time from injury to first aid	<6 h	31 (48.44)	28 (50.00)	0.029	0.864
	≥6 h	33 (51.56)	28 (50.00)		
Intracranial infection	Yes	16 (25.00)	27 (48.21)	7.000	0.008
	No	48 (75.00)	29 (51.79)		
Depression	Yes	6 (9.38)	31 (55.36)	29.611	<0.001
	No	58 (90.62)	25 (44.64)		
Anxiety	Yes	10 (15.63)	36 (64.29)	29.917	<0.001
	No	54 (84.37)	20 (35.71)		

Note: PTSD, post-traumatic stress disorder; TBI, traumatic brain injury; BMI, body mass index.

### Serum TMAO Levels in TBI Patients With PTSD

**Fig. 2.**
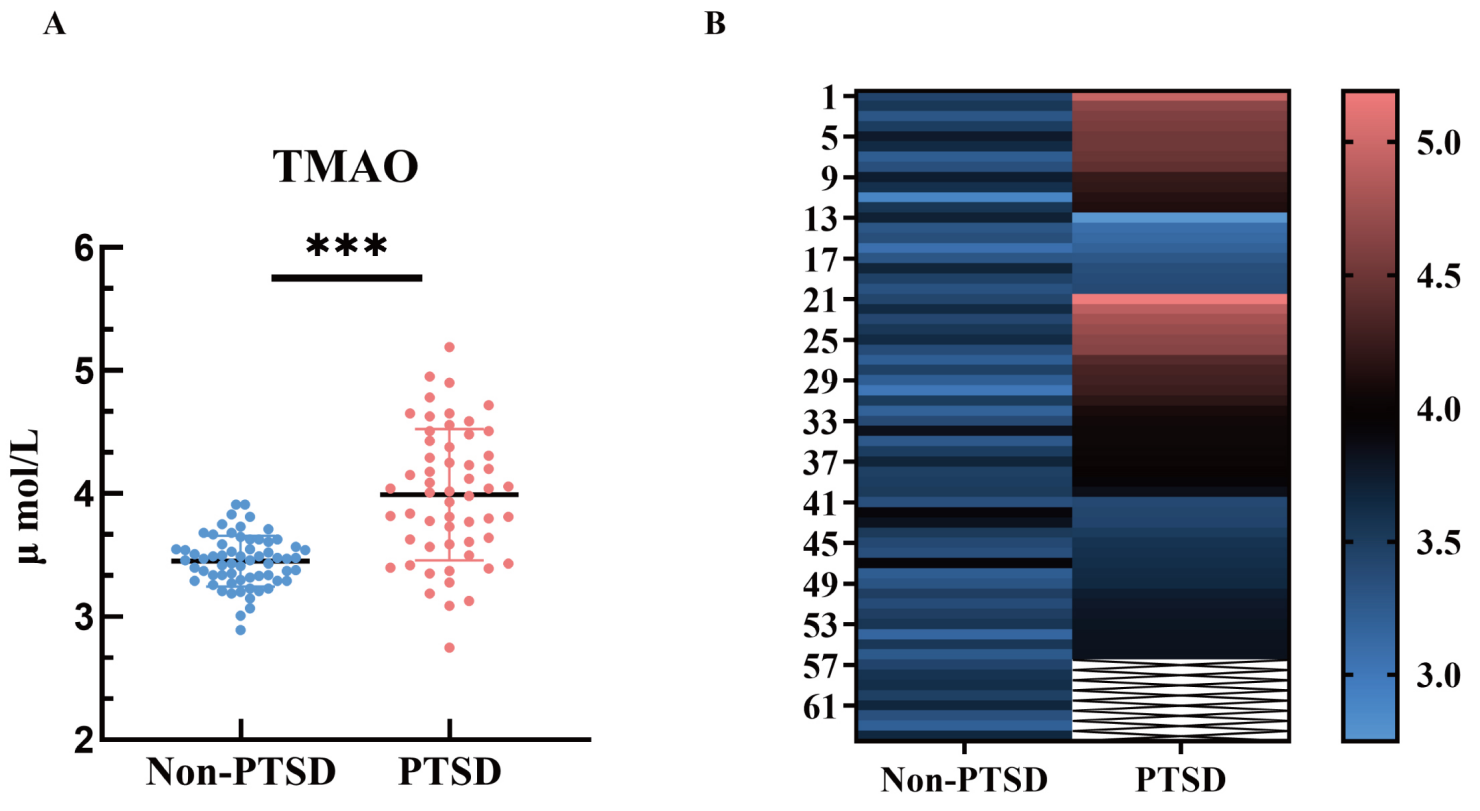
**Serum TMAO levels TBI Patients with PTSD**. (A) Scatter plot of serum TMAO values in the two groups. (B) Heat map of TMAO values in the serum of the two groups of patients. ^*⁣**^ indicates a comparison with the non-PTSD group, *p* < 0.001.

Serum TMAO levels were evaluated in both groups. The non-PTSD group had a serum TMAO level of 3.45 ± 0.21, while the PTSD group exhibited a level of 3.99 ± 0.53. The TMAO levels were significantly higher in the PTSD group than those in the non-PTSD group (*p* < 0.001, Table 2, Fig. 2).

**Table 2.** **Serum TMAO levels in TBI patients with PTSD**.

Variables	Non-PTSD group	PTSD group	*t*	*p*-value
N (%)	64 (53.33%)	56 (46.67%)		
TMAO (µmol/L)	3.45 ± 0.21	3.99 ± 0.53	7.516	<0.001

TMAO, trimethylamine N-oxide.

### Logistic Multivariate Regression Analysis of Risk Factors for PTSD Comorbid With TBI

The factors with significant differences in univariate analysis were sequentially assigned and included in multivariate analysis. The assignments were as follows: gender (“female” = 0, “male” = 1), per capita monthly household income (“<$275” = 0, “≥$275” = 1), financial reimbursement (“no” = 0, “yes” = 1), primary caregiver (“custody of kin” = 1, “unrelated care” = 0), intracranial infection (“no” = 0, “yes” = 1), depression (“no” = 0, “yes” = 1), anxiety (“no” = 0, “yes” = 1), and TMAO (“<3.57” = 0, “≥3.57” = 1).

Logistic regression analysis revealed female gender, low per capita monthly household income, depression, anxiety, and serum TMAO levels as significant risk factors for PTSD in TBI patients (Table 3). Among these factors, gender (odds ratio (OR) = 0.050, 95% confidence interval (CI): 0.011–0.241, *p* <0.001) and serum TMAO levels (OR = 24.505, 95% CI: 5.162–116.337, *p* < 0.001) were the most significant predictors. Additionally, depression (OR = 5.162, 95% CI: 1.002–26.578, *p* = 0.049), anxiety (OR = 10.914, 95% CI: 2.238–53.222, *p* = 0.003), and per capita monthly household income (OR = 0.143, 95% CI: 0.033–0.624, *p* = 0.010) substantially contributed to PTSD development. In contrast, intracranial infection (OR = 2.116, 95% CI: 0.547–8.810, *p* = 0.227), financial reimbursement (OR = 0.684, 95% CI: 0.185–2.536, *p* = 0.570), and primary caregiver (OR = 0.232, 95% CI: 0.051–1.068, *p* = 0.061) did not significantly contribute to PTSD risk (Table 3).

**Table 3.** **Logistic multivariate regression analysis of risk factors for PTSD comorbid with TBI**.

Variables	β	SE	Wald $\chi^{2}$ value	*p*-value	OR (95% CI)
Constant	0.186	0.989	0.035	0.851	1.204
Gender	–2.986	0.799	13.983	<0.001	0.050 (0.011–0.241)
Per capita monthly household income	–1.947	0.752	6.697	0.010	0.143 (0.033–0.624)
Financial reimbursement	–0.379	0.668	0.322	0.570	0.684 (0.185–2.536)
Primary caregiver	–1.459	0.778	3.519	0.061	0.232 (0.051–1.068)
Intracranial infection	0.749	0.690	1.180	0.227	2.116 (0.547–8.810)
TMAO	3.199	0.795	16.203	<0.001	24.505 (5.162–116.337)
Depression	1.641	0.836	3.853	0.049	5.162 (1.002–26.578)
Anxiety	2.390	0.808	8.741	0.003	10.914 (2.238–53.222)

Note: SE, standard error; OR, odds ratio; CI, confidence interval.

### Univariate Analysis of Risk Factors for Severe PTSD Comorbid With TBI 

Analysis of the clinical data revealed significant differences between the mild PTSD and severe PTSD groups concerning several key factors. Specifically, per capita monthly household income (*p* = 0.038), primary caregiver status (*p* = 0.038), intracranial infection (*p* = 0.017), depression (*p* = 0.004), and anxiety (*p* = 0.008) showed significant associations with the severity of PTSD in TBI patients. Interestingly, patients with a per capita monthly household income below $275 had a higher likelihood of severe PTSD (*p* = 0.038). Similarly, patients with higher depression (*p* = 0.004) and anxiety (*p* = 0.008) were more likely to experience severe PTSD. In contrast, intracranial infections (*p* = 0.017) and unrelated primary caregivers(*p* =0.038), were more prevalent in the mild PTSD group.

Notably, the two groups exhibited no significant differences regarding gender, age, BMI, marital status, type of injury (closed vs. open head injury), or the time from injury to first aid (all *p* > 0.05) (Table 4).

**Table 4.** **Univariate analysis of risk factors for severe PTSD comorbid with TBI [$\bar{x}$ ± s, n (%)]**.

Variables		Mild PTSD	Severe PTSD	*t*/$\chi^{2}$	*p*-value
Gender	Male	7 (26.92)	13 (43.33)	1.634	0.201
	Female	19 (73.08)	17 (56.67)		
Age (years)		45.42 ± 4.04	46.33 ± 4.47	0.795	0.430
BMI (kg/m^2^)		22.58 ± 3.08	22.09 ± 2.74	0.645	0.522
Marital status	Married	19 (73.08)	16 (53.33)	2.317	0.128
	Unmarried, divorced and	7 (26.92)	14 (46.67)		
Per capita monthly household income	<$275	13 (50.00)	23 (76.67)	4.314	0.038
	≥$275	13 (50.00)	7 (23.33)		
Injury factors	Traffic accident	12 (46.15)	13 (43.33)	1.428	0.699
	Fall down	6 (23.08)	9 (30.00)		
	Violence	6 (23.08)	4 (13.33)		
	Other	2 (7.69)	4 (13.33)		
Type of damage	Closed head injury	12 (46.15)	14 (46.67)	0.001	0.969
	Open head injury	14 (53.85)	16 (53.33)		
Financial reimbursement	Yes	10 (38.46)	12 (40.00)	0.014	0.906
	No	16 (61.54)	18 (60.00)		
Primary caregiver	Custody of kin	13 (50.00)	23 (76.67)	4.314	0.038
	Unrelated care	13 (50.00)	7 (23.33)		
Time from injury to first aid	<6 h	12 (46.15)	16 (53.33)	0.287	0.592
	≥6 h	14 (53.85)	14 (46.67)		
Intracranial infection	Yes	17 (65.38)	10 (33.33)	5.731	0.017
	No	9 (34.62)	20 (66.67)		
Depression	Yes	9 (34.62)	22 (73.33)	8.449	0.004
	No	17 (65.38)	8 (26.67)		
Anxiety	Yes	12 (46.15)	24 (80.00)	6.950	0.008
	No	14 (53.85)	6 (20.00)		

### Serum TMAO Levels in Severe PTSD Patients Comorbid With TBI

**Fig. 3.**
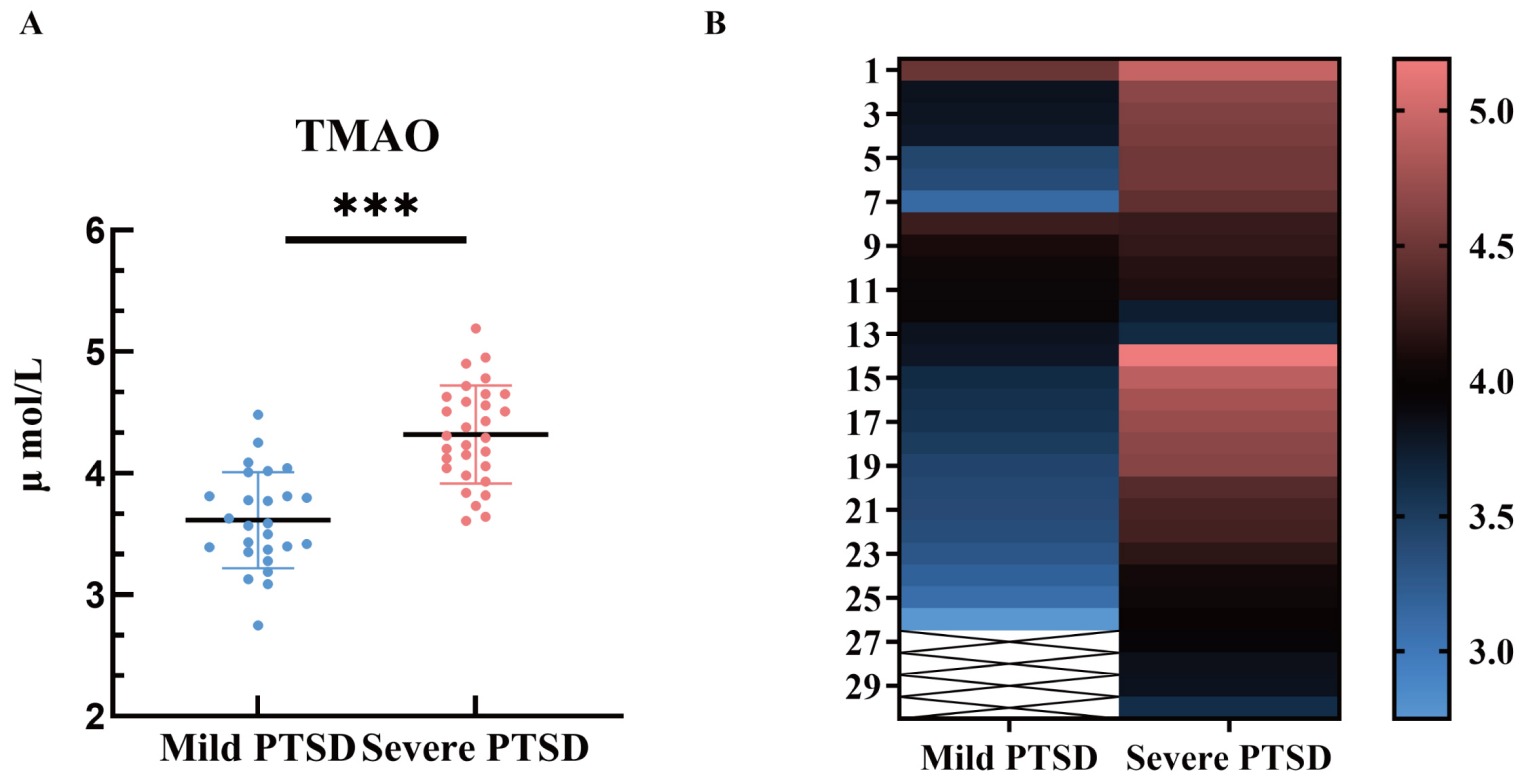
** Serum TMAO levels in severe PTSD patients comorbid with TBI**. (A) Scatter plot of serum TMAO levels in the two groups. (B) Heat map of TMAO levels in the serum of the two groups of patients. ^*⁣**^ indicates a comparison with the non-PTSD group, *p* < 0.001.

To investigate the differential expression of serum TMAO levels, we analyzed both mild and severe PTSD patient groups. The results showed that the TMAO levels in severe PTSD patients comorbid with TBI (4.31 ± 0.40 µmol/L) were significantly higher than those with mild PTSD (3.61 ± 0.40 µmol/L), with a statistically significant difference (*p* < 0.001). This finding suggests a significant association between TMAO expression and the severity of PTSD in TBI patients (Table 5, Fig. 3).

**Table 5.** **Serum TMAO levels in severe PTSD patients comorbid with TBI**.

Variables	Mild PTSD	Severe PTSD	*t*	*p*-value
N (%)	26 (46.43)	30 (53.57)		
TMAO (µmol/L)	3.61 ± 0.40	4.31 ± 0.40	6.593	<0.001

### Multivariate Analysis of Risk Factors for Severe PTSD Comorbid With TBI

Factors with significant differences identified in the univariate analysis were coded and included in the multivariate analysis. The factors were coded as follows: monthly per capita family income (“<$275” = 0, “≥$275” = 1); primary caregiver (“non-relative” = 0, “relative” = 1); intracranial infection (“No” = 0, “Yes” = 1); depression (“No” = 0, “Yes” = 1); anxiety (“No” = 0, “Yes” = 1); serum TMAO level (“<3.81” = 0, “≥3.81” = 1).

Logistic regression analysis identified per capita monthly household income, intracranial infection, depression, anxiety, and serum TMAO levels as significant risk factors for PTSD in TBI patients. Notably, higher per capita monthly household income (≥$275) (OR: 0.063, 95% CI: 0.009–0.421, *p* = 0.004) and intracranial infection (OR: 0.090, 95% CI: 0.015–0.557, *p* = 0.010) were associated with a decreased risk of severe PTSD. In contrast, depression (OR: 6.720, 95% CI: 1.188–37.997, *p* = 0.031), anxiety (OR: 5.684, 95% CI: 1.038–31.124, *p* = 0.045), and elevated serum TMAO levels (OR: 7.099, 95% CI: 1.072–47.028, *p* = 0.042) were strongly associated with a higher risk of severe PTSD. These findings highlight the multifactorial nature of PTSD risk following TBI (Table 6).

**Table 6.** **Multivariate analysis of risk factors for severe PTSD comorbid with TBI**.

Variables	β	SE	Wald $\chi^{2}$ value	*p*-value	OR (95% CI)
Constant	–1.057	0.923	1.311	0.252	0.347
Per capita monthly household income	–2.771	0.973	8.113	0.004	0.063 (0.009–0.421)
Primary caregiver	0.964	0.940	1.052	0.305	2.623 (0.415–16.561)
Intracranial infection	–2.404	0.928	6.717	0.010	0.090 (0.015–0.557)
Depression	1.905	0.884	4.645	0.031	6.720 (1.188–37.997)
Anxiety	1.738	0.867	4.013	0.045	5.684 (1.038–31.124)
TMAO	1.960	0.965	4.128	0.042	7.099 (1.072–47.028)

### The Diagnostic Value and Correlation of TMAO With PTSD Severity Comorbid With TBI

**Fig. 4.**
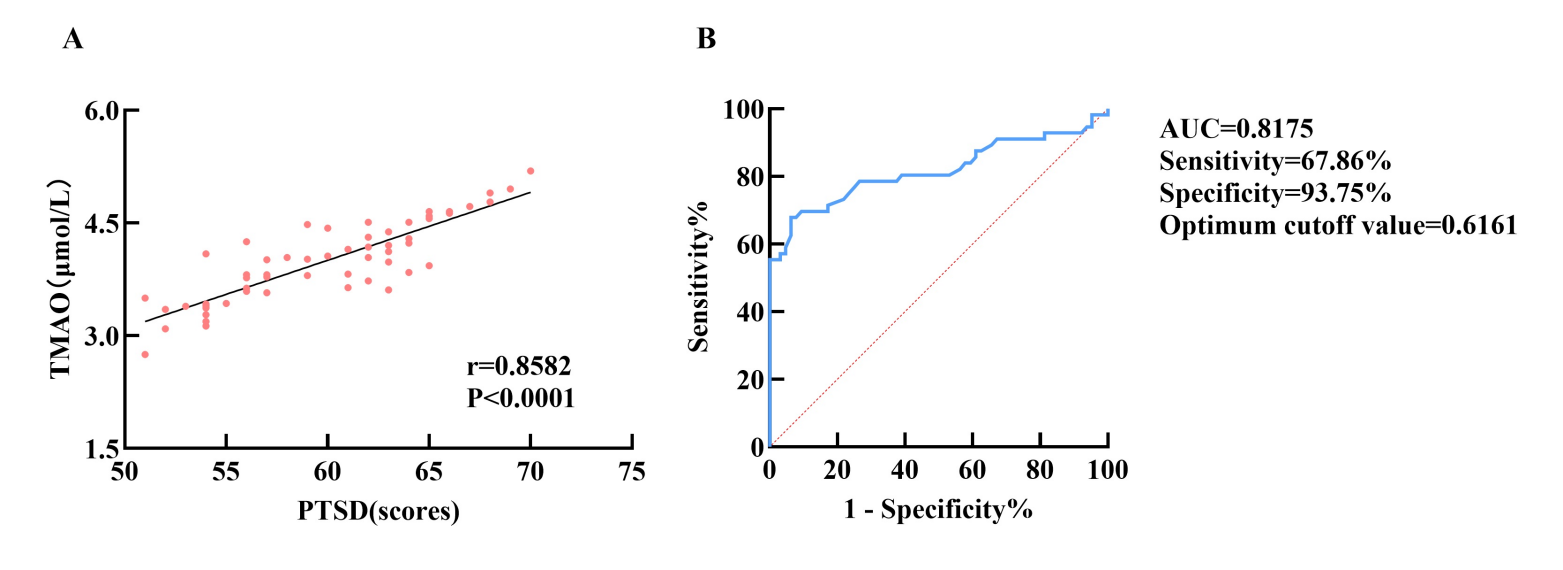
**The diagnostic value and correlation of TMAO with PTSD severity comorbid with TBI**. (A) A correlation between TMAO and PTSD severity. (B) ROC curve analysis of TMAO’s diagnostic efficacy for PTSD.

Correlation analyses revealed a moderate association between serum TMAO levels and PTSD-SS scores in the PTSD group (r = 0.8582, *p* < 0.0001). ROC curve analysis indicated that serum TMAO levels could be a predictive marker for PTSD risk in TBI patients, yielding an area under the curve (AUC) of 0.8175, a sensitivity of 67.86%, a specificity of 93.75%, and an optimal cutoff value of 0.6161 (Fig. 4).

## Discussion

TBI is a brain dysfunction caused by external mechanical forces, leading to acute and chronic neurological impairments. The underlying pathological mechanisms are complex, involving primary and secondary injuries. Primary injury occurs when an external force directly damages brain tissue [16]. Secondary injury is associated with neural responses after injury, blood-brain barrier disruption, and oxidative stress in the central nervous system [17]. Studies have indicated that TBI triggers structural changes in the central nervous system and as well as leads to various cognitive, emotional, and behavioral disorders, such as PTSD and depression, which significantly affect patients’ quality of life [18, 19].

In this study, the incidence of PTSD among TBI patients was 46.67%, with severe PTSD accounting for 21.67%. Furthermore, multivariate analysis identified female gender, lower monthly per capita family income, anxiety, depression, and increased serum TMAO levels as risk factors for PTSD. Specifically, lower monthly per capita family income, anxiety, depression, and serum TMAO levels were found to be risk factors for severe PTSD. However, intracranial infections were found to have a protective effect against severe PTSD. These impacts may be due to immune responses triggered by infections that alter neuroinflammation, potentially reducing PTSD symptoms. Additionally, such infections may enhance brain adaptability and greater psychological resilience, helping individuals cope with trauma. However, this finding might be influenced by small sample sizes, necessitating further research to confirm these outcomes. These observations underscore the complex interaction between socioeconomic and biological factors in the development of PTSD following TBI. Gender differences, particularly the higher susceptibility of women to PTSD following trauma [20, 21], and the crucial role of income and social support in psychological stress response and resilience were also highlighted.

The association between PTSD and TBI has been extensively studied, with evidence indicating that PTSD is substantially more prevalent in TBI patients compared to the general population. This increased incidence may be attributed to shared physiological mechanisms, including neuroinflammation [22, 23, 24], dysregulation of the hypothalamic-pituitary-adrenal (HPA) axis [25, 26], and altered neurotransmitter signaling. Additionally, TBI also sensitizes neural circuits involved in fear and anxiety processing, thereby intensifying stress responses. Structural brain changes associated with TBI, such as white matter damage and hippocampal atrophy [27], further increase PTSD risk. The severity of TBI may influence PTSD risk. Notably, PTSD rates tend to be higher in mild TBI cases, which is a common type of brain injury in both military and civilian trauma [28]. However, the underlying mechanisms linking TBI and PTSD are complex, underscoring the need for further exploration into potential biomarkers for early detection and monitoring of PTSD. Although TBI severity is known to impact on long-term outcomes, our study did not assess this factor due to data constraints. Therefore, the potential influence of TBI severity on PTSD outcomes remains speculative and requires further investigation.

The human gut microbiota and its metabolites impact central nervous system function beyond their role in digestion and absorption. Research has shown that changes in gut microbiota after brain injury can significantly impact patient recovery [29]. The gut microbiota produces a key metabolite, TMAO, which plays a crucial role in cardiovascular and metabolic diseases and is closely associated with peripheral artery disease, cancer, and central nervous system disorders. For instance, a study by Arrona *et al*. [30] found a strong correlation between increased TMAO levels and Alzheimer’s disease (AD). Similarly, Zhou *et al*. [31] reported significantly elevated serum TMAO levels in Parkinson’s disease patients, suggesting that TMAO may impact the development and progression of the disease. These findings indicate that TMAO can affect central nervous system responses by affecting gut-brain axis function and could exacerbate stress responses after brain injury by compromising the blood-brain barrier.

Our study indicated that TBI patients with PTSD exhibit higher serum TMAO levels compared to those without PTSD, highlighting a potential role of TMAO in the pathophysiology of PTSD following TBI. Previous research has linked TMAO levels to PTSD after acute myocardial infarction [32]. While these findings suggest TMAO’s involvement in cardiovascular-related PTSD, our study focuses on PTSD comorbid with TBI, which involves distinct mechanisms like neuroinflammation and brain injury. Therefore, while both studies explore TMAO’s role in PTSD, our research offers new insights into its specific role in PTSD associated with TBI. These observations highlight the need for further exploration into TMAO’s differential effects across various PTSD types.

Additionally, the study also revealed a positive relationship between TMAO levels and PTSD severity. We hypothesize that TMAO may influence the onset and progression of PTSD following TBI for several reasons. First, TMAO has been shown to activate multiple signaling pathways, particularly after nervous system injury, where the release of pathway factors can further exacerbate neuronal damage. Second, TMAO may increase brain tissue injury through oxidative reactions. Lastly, TMAO can compromise the integrity of the blood-brain barrier, allowing more neurotoxins and harmful factors to enter brain tissue, leading to central nervous system dysfunction and promoting the development of PTSD after TBI. Furthermore, ROC curve analysis indicated a specificity of 93.75% for TMAO, with a high AUC of 0.8175, suggesting TMAO’s potential as a biomarker for PTSD incidence.

In summary, the significant association between TMAO and PTSD in TBI patients indicates that it could serve as a novel biomarker for detecting and preventing mental disorders. Future research should focus on more extensive cohort studies to explore the comprehensive role of TMAO and its associated pathways in PTSD and other neuropsychiatric disorders, potentially providing new scientific evidence for optimizing clinical treatment strategies.

Despite some promising findings, this study has some limitations. Firstly, the sample size was relatively small and obtained from a single hospital, which may not fully represent the broader population of TBI patients. Additionally, the assessment of PTSD was based on PTSD-SS [13], which may be subjected to self-report bias. The predictive value of TMAO for PTSD and its potential as a biomarker for early intervention needs to be further studied in larger, more diverse cohorts with long-term follow-up periods.

## Conclusion

This study explores the potential role of TMAO in PTSD comorbid with TBI, highlighting its significance as a biomarker in assessing the risk of PTSD. Future studies should validate its diagnostic and predictive value in patients with PTSD following TBI and explore approaches for regulating TMAO levels, providing new therapeutic strategies for clinical practice.

## Availability of Data and Materials

The data used and/or analyzed during the current study are available from the corresponding authors.
